# Epigenetic regulation of gene expression in cancer: techniques, resources and analysis

**DOI:** 10.1093/bfgp/elx018

**Published:** 2017-08-11

**Authors:** Luciane T Kagohara, Genevieve L Stein-O’Brien, Dylan Kelley, Emily Flam, Heather C Wick, Ludmila V Danilova, Hariharan Easwaran, Alexander V Favorov, Jiang Qian, Daria A Gaykalova, Elana J Fertig

**Keywords:** epigenetics, cancer, bioinformatics, methylation, chromatin, data integration

## Abstract

Cancer is a complex disease, driven by aberrant activity in numerous signaling pathways in even individual malignant cells. Epigenetic changes are critical mediators of these functional changes that drive and maintain the malignant phenotype. Changes in DNA methylation, histone acetylation and methylation, noncoding RNAs, posttranslational modifications are all epigenetic drivers in cancer, independent of changes in the DNA sequence. These epigenetic alterations were once thought to be crucial only for the malignant phenotype maintenance. Now, epigenetic alterations are also recognized as critical for disrupting essential pathways that protect the cells from uncontrolled growth, longer survival and establishment in distant sites from the original tissue. In this review, we focus on DNA methylation and chromatin structure in cancer. The precise functional role of these alterations is an area of active research using emerging high-throughput approaches and bioinformatics analysis tools. Therefore, this review also describes these high-throughput measurement technologies, public domain databases for high-throughput epigenetic data in tumors and model systems and bioinformatics algorithms for their analysis. Advances in bioinformatics data that combine these epigenetic data with genomics data are essential to infer the function of specific epigenetic alterations in cancer. These integrative algorithms are also a focus of this review. Future studies using these emerging technologies will elucidate how alterations in the cancer epigenome cooperate with genetic aberrations during tumor initiation and progression. This deeper understanding is essential to future studies with epigenetics biomarkers and precision medicine using emerging epigenetic therapies.

## Introduction

Cancer is a complex disease. The malignant transformation is a multistep process associated with the accumulation of numerous molecular alterations. These molecular changes impact cellular function within the tumor and its microenvironment, and culminate in the hallmarks of cancer: sustained proliferative signaling, resistance to apoptosis, senescence, angiogenesis, invasion and metastasis, deregulating cellular energetics, avoiding immune destruction, tumor-promoting inflammation, and genome instability and mutation [1]. Numerous genetic alterations (mutations, loss of heterozygosity, deletions, insertions, aneuploidy, etc.) have been associated with carcinogenesis [2] and can sometimes present a clear oncogenic function being considered as cancer driver mutations in such cases [3]. All these genetic alterations ultimately result in aberrant gene expression. However, the landscape of genetic alterations is insufficient to explain the pervasive gene expression changes and alterations to cellular function in cancer [4]. For example, according to the Knudson two-hit hypothesis deletions on both alleles of tumor suppressor genes block the mechanisms in the cell that prevent aberrant cellular growth [5, 6]. In many cancers, one of these alterations (or ‘hits’) is a hereditary or somatic mutation in a tumor suppressor gene and the second ‘hit’ is an acquired mutation or copy number loss in the other allele. Sometimes, the second genetic ‘hit’ is not observed. Instead, epigenetic alterations cause the changes in gene expression of tumor suppressors in place of genetic changes [4].

Epigenetic alterations are heritable traits that impact the phenotype by interfering with gene expression independent of the DNA sequence [7, 8]. These epigenetic mechanisms include DNA methylation, chromatin remodeling, noncoding RNAs (ncRNAs), binding of regulatory proteins [such as 11-zinc finger protein (CTCF) and brother of the regulator of the imprinted site (BORIS)], and transcription factors [9, 10]. Many of these mechanisms also affect chromatin states. Epigenetic changes are as pervasive in cancer as genetic alterations, and likely are responsible for the hidden source of variation in cancer [4].

Epigenetic events can also act concomitantly with other molecular processes in normal states or disease to have persistent gene expression and functional alterations. For example, epigenetic alterations that impact transcription factor binding can explain genome-wide transcription dysregulation independent of genetic variation in cancer [11]. Recent studies have also found that cancer mutations are associated with the chromatin structure of the tissue of origin [12]. These mutations occur more frequently in regions of closed chromatin, which are inaccessible to DNA repair genes during replication [12]. Therefore, alterations to chromatin structure may be critical drivers of cancer.

Epigenetic alterations with functional impacts on gene expression in individual tumor remain key targets of interest [9, 13–15]. Notably, emerging epigenetic therapies can revert specific epigenetic alterations in cancer [9, 13–15]. There are two groups of drugs used for epigenetic therapy: (1) DNA methyltransferase inhibitors (DNMTi) and histone deacetylase inhibitors (HDACi); (2) targeted therapeutic agents to block specific genes that when mutated cause deregulation of epigenetic markers. Both classes of inhibitors cause pervasive genome-wide epigenetic changes. There are currently numerous ongoing clinical trials using these inhibitors to treat different tumor types. The FDA has already approved some of these agents to treat cancer patients. The mechanisms of action and the list of approved epigenetic drugs or on clinical trials were previously reviewed in detail [16, 17]. Development of therapeutic agents to reverse specific alterations to the epigenetic landscape is currently an active area of research. Therefore, genome-wide characterization of epigenetic aberrations is crucial for the development of new therapies and to identify new biomarkers for existing epigenetic inhibitors [16, 17].

**Figure 1.**
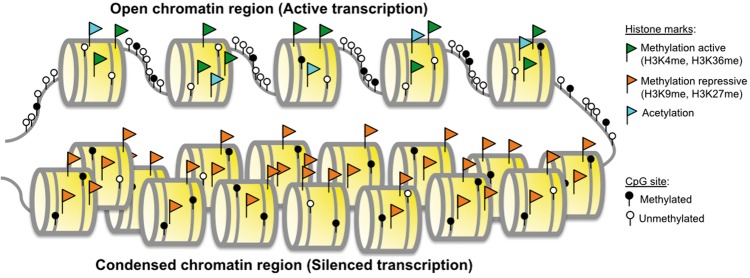
Epigenetic mechanisms of gene expression regulation. Gene transcription occurs in regions, where the chromatin conformation is more open (active chromatin regions). Transcriptionally silenced genes are found in regions with compact chromatin. Active chromatin areas are characterized by unmethylated DNA CpG sites, histone acetylation and histone active markers, such as H3K4 and H3K36 methylation. Transcriptional silencing is characterized by methylated CpG sites, histone deacetylation and repressive histone markers. These epigenetic alterations occur in areas of condensed chromatin, in which nucleosomes are positioned close to each other. In these regions, the DNA structure and protein complexes that regulate compact confirmation block DNA accessibility to transcription factors resulting in epigenetic silencing. (A colour version of this figure is available online at: https://academic.oup.com/bfg)

This review focuses on describing current resources to discover reversible epigenetic events in cancer that may be targeted therapeutically in cancer: DNA methylation and chromatin organization (Figure 1). We describe techniques, model systems, and data resources with high-throughput epigenetic data in cancer. We also discuss the emerging bioinformatics algorithms for analysis of these data and their integration with high-throughput transcriptional data in cancer. The review focuses on experimental and computational techniques for cancer epigenetics data that is currently available in the literature. Therefore, this review summarizes the experimental and computational tools that can find the hidden sources of genetic variation in cancer and for precision medicine of novel epigenetic therapies from current publicly available data.

## Reversible epigenetic events

### DNA methylation

DNA methylation is the addition of a methyl radical (CH_3_) to the 5-carbon on cytosine residues (5mC) in CpG dinucleotides [18–20]. Modifications to DNA methylation are normal events that have critical roles during different stages of human development, silencing of genome repetitive elements, protection against the integration of viral sequences, genomic imprinting, X chromosome inactivation in females and transcriptional regulation [7, 20, 21]. CpG methylation differs between tumor and normal tissues. Removal of CpG methylation specific to cancer cells is referred to as hypomethylation, and CpG methylation specific to cancer cells is referred to as aberrant methylation or hypermethylation.

DNA methylation changes to regulatory elements (promoters; insulators; enhancers) with CpG-concentrated regions known as CpG islands (CpGIs) have been a focus within genome-wide epigenetic studies. These CpGIs are long sequences (∼800 nucleotides in average, range 200–10 000) that have a high concentration of CpGs (∼10%) and C + G content (>55%) in comparison with the rest of the genome (1% of CpG and 42% C + G content) [22, 23]. DNA methylation changes outside of CpGI regions or their ‘CpG shores’ are also apparent. The methylation patterns in these shores are highly tissue specific in normal human samples [24]. The alterations in DNA methylation in cancer extend well beyond CpGI shores [24, 25]. The methylation profiles in tumor samples at some regions distant from CpGIs may be more similar to other normal tissues than the cell of origin [24]. The impact and function of these distant alterations remain poorly understood.

Genome-wide hypomethylation was the first described epigenetic aberration in cancer [26]. Loss of normal DNA methylation levels is associated with genome instability and aneuploidy, reactivation of transposable elements and loss of imprinting [26, 27]. Nonetheless, hypermethylation is investigated more frequently. This event is associated with gene silencing [28–30] by (1) blocking transcription directly, by blocking transcription factors binding to their specific sites; or indirectly by (2) recruiting of protein complexes with high affinity for methylated DNA (methyl-binding domain complexes, MBDs) (Figure 1) [18, 19, 21, 29, 31–34]. Aberrant DNA methylation can silence tumor suppressor genes as effectively as inactivating mutations. Therefore, hypermethylation can serve as one of the hits required for oncogenesis described in Knudson’s two-hit hypothesis [4, 5, 35–37]. Hypermethylation of gene promoters is not limited to protein-coding genes. DNA methylation also regulates the expression of noncoding RNAs, some of which have a role in malignant transformation [4].

Further epigenetic changes by TET (ten-eleven translocation) 5mC dioxygenases revert DNA methylation in cancer. Members of the TET family are capable of oxidizing 5mC, causing a conversion to 5-hydroxymethylcytosine (5hmC). These same proteins can subsequently oxidize 5hmC to 5-formylcytosine (5fC) and 5-carboxycytosine (5caC). The presence of these oxidative 5mC products is associated with gene expression restoration and is considered an active mechanism of methylation reversion [38, 39]. Studies to determine the role of cytosine variants in cancer are emerging in the literature. The development of techniques to characterize 5hmC, 5fC and 5caC are relatively recent, which explains the lack of high-throughput methods for genome-wide mapping. Moreover, no therapeutics that can currently target DNA methylation reversions by TET family members.

### Chromatin modifications

Histone marks are posttranslational modifications of core histone proteins that affect chromatin structure [40]. These modifications correlate with open or closed conformations of chromatin and drive differential access of genes to transcription factors and regulatory proteins (Figure 1). Therefore, cells can use histone alterations as facile to regulate their gene expression dynamically. They are frequently deregulated in complex diseases, including cancer [4]. Changes in chromatin landscape co-occur with alterations of DNA methylation. Histone methylation (20 variants) and acetylation (18 variants) are the most common modifications that primarily target lysine residues of histone tails [41]. Many more modifications exist, including phosphorylation, ubiquitylation and glycosylation, which spread to other amino acid residues, such as arginine, serine and threonine [42–45]. Critical amino acids of histone tails are frequent targets of epigenetic modifications. Genes encoding for these amino acids in these tails are also frequently mutated in cancers [46–48]. Multiple regulatory proteins write, read and erase histone marks [49]. Many of these proteins are also dysregulated in diseased cells [50]. Because histone marks have stable covalent structures, they can be inherited during cell division and DNA duplication and serve as disease markers [51]. Analysis of chromatin structure and its regulatory machinery is critical to developing epigenetics therapies.

Histone modifications result from activity in three groups of proteins: epigenetic writers, such as histone acetyltransferases (HATs), histone methyltransferases (HMTs), protein arginine methyltransferases (PRMTs) and kinases; epigenetic readers, such as proteins containing bromodomains and chromodomains; and epigenetic erasers, such as histone deacetylases (HDACs), lysine demethylases (KDMs) and phosphatases [52–54]. These proteins alter chromosomal structure by directly modifying and regulating DNA accessibility. Genetic alterations to genes encoding for histone modifying proteins can cause widespread epigenetic changes. These changes can dysregulate cell homeostasis pathways independent of mutations to critical oncogenes or tumor suppressor genes in these pathways [55]. Mutations in the histone modifiers and readers of protein-coding genes occur frequently in all cancer types. Therefore, integrated analyses of genome-wide genetic and epigenetic studies are essential to determine the source of alterations to the distribution of histone markers [56, 57]. A recent study by Huether *et al.* found that the frequency of mutations in histone modifying genes varies by tumor type, occurring with highest frequency in brain tumors and leukemias (30% of the cases). The frequency and type of alterations to histone modifying genes vary within tumor subtypes, with histone H3 mutations present in almost half of high-grade gliomas [58]. Other tumor types, such as esophageal squamous cell carcinomas, bladder cancer, medulloblastoma and lung cancer, also have frequent mutations in epigenetic modifier genes. However, these studies lack functional validation of the role of genetic alterations to histone modifying genes to chromatin structure and in cancer progression [59–62]. These mutations will alter the epigenetic landscape of tumors, which will subsequently impact sensitivity to epigenetic therapies. Therefore, refined analysis of mutations in histone modifying genes may provide new genetic biomarkers to predict patient response to epigenetic therapy.

Chromatin changes also result from expression changes to types of ncRNAs, transcripts that are not translated into proteins. Details of the role of ncRNAs in gene expression regulation are the subject of other reviews [63–65]. They describe all observed functions for ncRNA. One example described is binding of long noncoding RNAs (lncRNAs) bind to DNA and to chromatin remodeling complexes. Both cases are associated with complex alterations in the distribution of nucleosomes. The functional role of specific ncRNAs on cancer epigenetics is an active area of research.

## High-throughput platforms for epigenetic analysis

**Figure 2.**
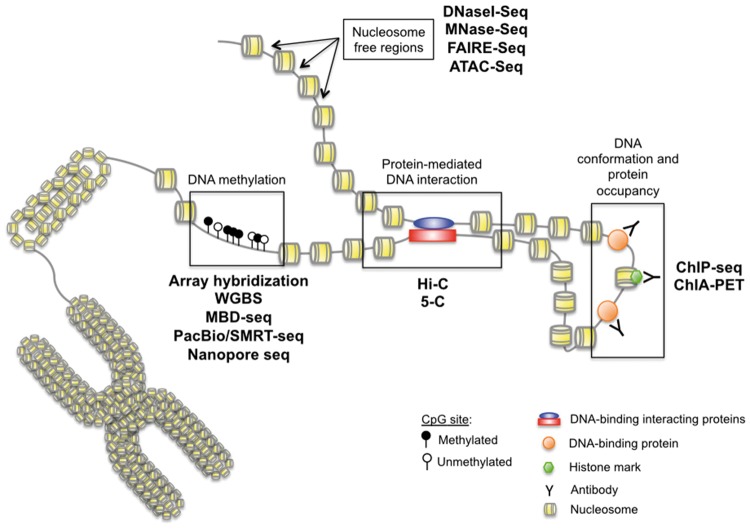
Epigenetics measurement techniques. A wide variety of methods characterize epigenetic alterations. Currently, the most common genome-wide approaches identify nucleosome-free regions (DNaseI-Seq; MNase-Seq; FAIRE-Seq; ATAC-Seq), protein-mediated DNA interaction sites (Hi-C; 5-C), histone marks and DNA-binding proteins (ChIP-Seq; ChIA-PET) and DNA methylation (array hybridization, WGBS, MBD-Seq, PacBio, nanopore). (A colour version of this figure is available online at: https://academic.oup.com/bfg)

In the current era of high-throughput data, new technologies to measure the genome-wide state of DNA methylation and chromatin structure are actively emerging (Figure 2). Following the history of the field, many measurement platforms were first developed in microarrays and adapted to next-generation sequencing technologies. As a result, cancer biologists have access to unprecedented measurement technologies that can assess genome-wide DNA methylation and chromatin modifications, accessibility, protein interactions and binding. Here, in this review, we will briefly describe high-throughput approaches for epigenetic mapping from DNA methylation, chromatin modification markers and chromatin structure commonly used in cancer genomics, with details about each measurement technology in Supplemental File 1.

### DNA methylation

Numerous microarray and sequencing-based technologies have been used to measure DNA methylation (Table 1). DNA methylation can be measured on native DNA through recognition of methylated cytosines by antibodies [methylated DNA immunoprecipitation (MeDIP)] or by conjugated methyl-CpG binding proteins (MBPs) [51]. The antibodies can also recognize DNA methylation-associated proteins such as MeCP2 with chromatin immunoprecipitation (ChIP)-based technologies, which can be used to estimate DNA methylation. Massively parallel next-generation sequencing and arrays to measure DNA methylation-enriched fragments provide whole-genome evaluation of DNA methylation with high resolution of mCpG sites. False negatives may arise from incomplete binding of the antibodies. Nonetheless, these techniques have strong true-positive rates because of the nanomolar binding affinity to symmetrically methylated CpG.

**Table 1.** High-throughput DNA methylation techniques [66–77]

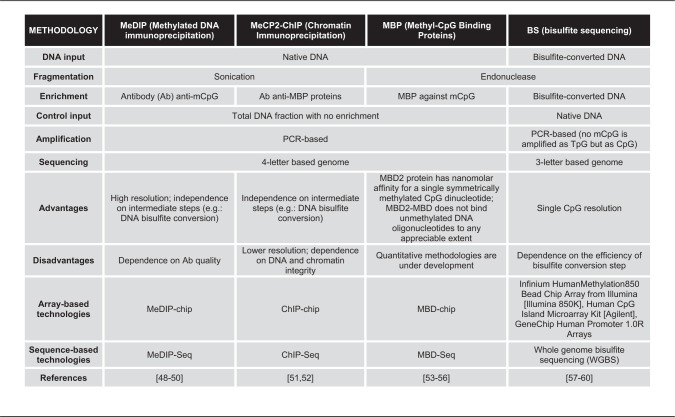

Whole-genome profiling of bisulfite converted DNA can also measure DNA methylation. Bisulfite treatment deaminates non-methylated cytosine to uracils to be further recognized as thymidine during sequencing or array-based probe annealing but leaves methylated cytosines stay unchanged. Both converted DNA and untreated input controls are profiled in arrays (Illumina HumanMethylation Bead Chip Arrays, Agilent Human CpG Island Microarray; and Affymetrix GeneChip Human Promoter 1.0R Arrays) or with next-generation sequencing (WGBS). In both cases, the ratio between methylated and unmethylated signals at each specific CpG is proportional to its methylation level. Bisulfite conversion reduces the genome from four nucleotides to three in unmethylated regions. This alteration makes alignment to the reference genome or annealing to the particular probe nonunique, and may cause technical errors in quantification of DNA methylation data. Therefore, new bioinformatics techniques for preprocessing bisulfite-based data remain a critical challenge preceding the analysis of DNA methylation data in cancer. In both cases, measurements of epigenetic variation within tumors are only possible with bisulfite sequencing techniques. Specifically, comparison of the epigenetic changes across reads can quantify the variation of epigenetic alterations in a single locus [78]. Single-cell bisulfite sequencing technologies are emerging to further refine the variability of the epigenetic landscape within tumor samples.

Additional methods are also developing to address the challenges of aligning bisulfite-converted DNA, including PacBio or single-molecule real-time (SMRT) sequencing and nanopore sequencing. PacBio uses fluorescent nucleotides to capture the signal during DNA replication. This technology measures a specific fluorescent signal that is generated for each nucleotide, and the time between the incorporation of two bases is measured. The structural chemical changes in the cytosine variants provide them with distinct times of incorporation allowing their discrimination from regular cytosines. PacBio has a further advantage over WGBS, as it allows sequencing of long fragments (10–60 kb), albeit at high error rates (∼15%) [79].

Nanopore sequencing also takes advantage of the differences in the chemical structure among cytosine variants. In this case, changes in the ionic current are specific to both sequence and cytosine variant. The changes in current are measured based on the time for single-strand DNA fragments take to pass through the nanopores in a lipid membrane. The measurements of current are then converted to nucleotides, including annotations of cytosine variants. A further advantage of nanopore sequencing is that it does not require DNA amplification. Currently, nanopore sequencing provides lower coverage than next-generation sequencing technologies and is, therefore, limited to small genomes or small portions of complex genomes. Moreover, robust bioinformatics algorithms for DNA methylation analysis with nanopore sequencing are still under development for reliable and reproducible interpretation of the data [80, 81].

### Chromatin structure and interaction

Chromatin is the DNA–protein complex that compacts and protects the genomic DNA within the cellular nucleus and the carrier of epigenetic information, with techniques to measure its structure and interaction domains summarized in Table 2. The structure of chromatin can be evaluated by DNA accessibility with restriction reagents, such as DNaseI or MNase, or with assay for transposase-accessible chromatin (ATAC). DNA binding of selected proteins can also evaluate chromatin structure (ChIP; formaldehyde-assisted isolation of regulatory elements (FAIRE)]. Additionally, the relative proximity of distant DNA regions to each other (HiC and chromatin interaction analysis by paired-end tag sequencing (ChIA-PET)] defines the 3D structure of chromatin. Similar to DNA methylation profiles, arrays or sequencing measure the whole-genome structure of chromatin from fragmented and enriched DNA. The binding of DNA by histones or other proteins, such as enzymes or transcription factors, decreases the accessibility of such regions to restriction, shredding or transposase tagmentation activity. Therefore, the reads that remain represent the aspect of chromatin structure that the experimental process modifies.

**Table 2.** High-throughput chromatin organization techniques [82–100]

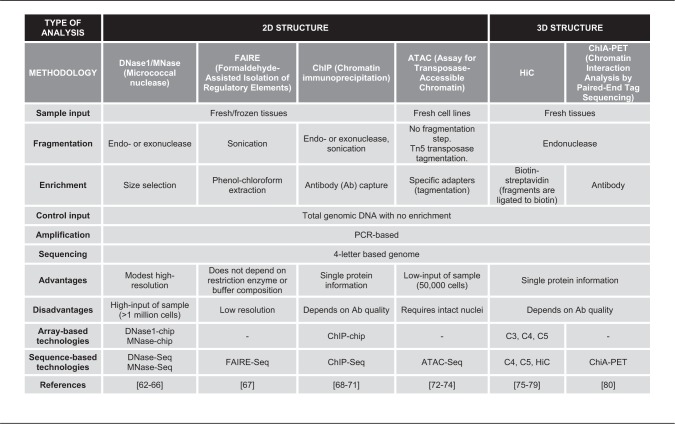

Chromatin state can be mainly classified into two types: active (or open) and inactive (or condensed). Whole-genome chromatin analysis reveals chromatin structure in regions of open chromatin, including intergenic regions and active regulatory elements, such as promoters, silencers and enhancers. Owing to the modest sequence dependence of enzymes like DNase1 or MNase, there is a probability of false-positive signals. Chromatin states can be further classified into more types such as promoter, enhancer and insulator. These chromatin states are often associated with different histone modifications. For example, H3K9ac is found in actively transcribed promoters, while H3K9me3 is found in constitutively repressed genes. Moreover, the combination of histone modifications can more precisely predict chromatin states. For instance, H3K4me and H3k27ac are marks for active enhancers. The histone modifications can be determined by ChIP-seq approach using antibodies against specific histone modifications. Public domain databases characterize histone antibody specificity to select optimal antibodies to measure histone modifications with ChIP [101].

Other restrictions of chromatin analysis arise from the requirement of high tissue input for most of the procedures. In addition, the chromatin integrity and DNA–protein binding strength highly depend on sample preservation, limiting analysis to fresh tissues or cell lines. Both of these restrictions prevent analysis of the majority of primary human tumors, which are small and sometimes not adequately preserved in tissue banks. Moreover, individual samples have different cellular properties that impact the chromatin digestion. The degree of chromatin digestion by endonucleases/exonucleases or by sonication must be empirically determined for each sample to avoid under- and over-digestion. Even on proper chromatin fragmentation, condensed chromatin with repressive marks can still be under-digested, resulting in the presence of longer DNA segments (>900 bp), poor amplification during library prep for the sequencing and low resolution of repressive histone marks. Both ChIP and ChIA-PET require further preparation of individual samples for each study protein. Therefore, analysis of several proteins can be costly and require a high volume of cells that is not available for most primary cancer samples.

## Model systems

**Figure 3.**
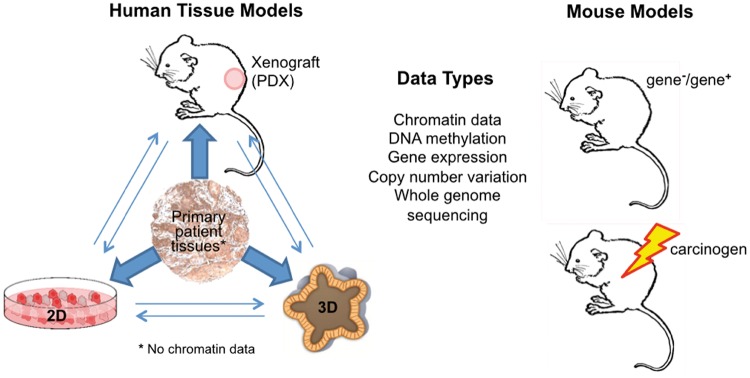
Epigenetic data can be obtained *in vivo* from primary tumor samples, PDXs and mouse models of cancer. While there are no limitations to measuring DNA methylation in patient samples, demands of high tissue quality and quantity limit measurements of chromatin structure and interaction data in patient samples. All epigenetic data can also be obtained *in vitro* with 2D (cancer cell lines) and 3D (organoids and conditionally reprogrammed cells) culture systems. (A colour version of this figure is available online at: https://academic.oup.com/bfg)

Ideally, the epigenetic measurement technologies will be applied to primary tumors samples to measure their epigenetic state. Such comprehensive profiling of DNA methylation has been performed extensively across measurement technologies. However, profiling chromatin in tumor samples is more challenging. Many chromatin assays require large quantities of high-quality DNA and the intact chromatin structure. However, tumor samples that are available for profiling are typically small and use preservation techniques that may degrade the quality of the DNA or chromatin structure. Therefore, both *in vitro* and *in vivo* model systems of cancer are used to determine the epigenetic state of many cancer types (Figure 3). Extending these techniques to humanized patient-derived xenografts (PDXs) is essential to determine the impact of the immune system to perform preclinical studies correlating the functional role of epigenetics on the efficacy of epigenetic inhibitors and immunotherapy.

## Epigenetics data sources

Numerous international high-throughput genomics databases from thousands of tumor samples and model systems are available in the public domain, reviewed in [102]. However, similar resources for epigenetics in cancer are still more limited. Recent efforts to organize and share large epigenomic data sets have created publicly available resources for discovery and validation in cancer. Cancer-specific resources range in specificity, including large, multi-assay data sets across epigenetics data modalities, such as DNA methylation, histones and chromatin structure. DNA methylation of 1001 cancer cell lines was measured with Illumina 450K arrays along with therapeutic sensitivity, copy number, somatic mutations and gene expression [103] and is freely available from the Gene Expression Omnibus (GEO Series GSE68379). Both The Cancer Genome Atlas (TCGA) and International Cancer Genome Consortium (ICGC) contain microarray measurements of DNA methylation in primary tumors and cancer models. DNA methylation of tumors has been assessed with sequencing technologies in isolated studies from smaller groups, with corresponding data often deposited into sources including GEO [104], dbGAP and ArrayExpress [105]. However, these sequencing-based DNA methylation data are available for fewer primary tumors than the array-based data from international consortia.

Currently, there are no databases of chromatin structure in primary tumors because of the dual challenge of sample quality and quantity described above. Therefore, projects such as ENCODE contain ChIP-seq data of histone marks and chromatin accessibility in numerous cancer cell lines in place of primary tumors [106]. Additional resources include FANTOM’s efforts to characterize promoter utilization in 250 cancer cell lines [107]. Similar to DNA methylation, chromatin measurements in other model organisms have been performed by individual laboratories. Data of chromatin structure of healthy samples from projects such as the Roadmap Epigenomics Project [108] have found unanticipated associations with mutations in cancer samples [109]. Therefore, comparison of epigenetics data in healthy tissue samples with cancer genomics data sets from distinct databases may also yield novel insights into the functional impacts of epigenetic regulation in cancer.

Determining the function of epigenetics alterations in cancer requires integration with genomics data. Ideally, these measurements would be made for the same tumor sample. Most prominently, TCGA and ICGC contain microarray measurements of DNA methylation, RNA-sequencing of transcription, reverse phase protein array (RPPA) for protein and phosphoprotein states, sequencing of mutations and copy number arrays in primary tumors. Availability of high-throughput genomics data ENCODE and FANTOM further enables correlation of chromatin features with gene expression to assess the functional changes resulting from epigenetic alterations in tumors or cancer cells and their association with DNA methylation. New resources containing all these sources for data for a wide range of cell lines and primary tissues are essential to establish epigenetic drivers and therapeutic targets in cancer.

### Resources to access, analyze and integrate epigenetics data

Despite the breadth of public domain epigenetics data sets, there is a lack of centralized resources to obtain the wide range of data from numerous studies in a centralized platform. There are several databases for chromatin data. For example, Cistrome is a centralized database of histone modifications [110], which includes Cistrome Cancer to integrate TCGA gene expression data with public ChIP-seq data to determine functional histone modifications in cancer. Another database, EnhancerAtlas (enhanceratlas.org), was specially designed for enhancer analysis and visualization across studies [111]. It contains enhancer annotation for >100 cell/tissue types, including both normal and cancer cells. The enhancer annotations are derived from multiple, independent experimental evidence including chromatin accessibility, histone modifications and enhancer RNAs (eRNAs). Furthermore, the database provides several analytic tools, so that the users can compare the enhancer activity across different cell types or connect the enhancers and target genes.

Genome browsers are tools to visualize and integrate data from publicly available repositories. Notable genome browsers include the UCSC Genome Browser [112, 113], the Ensembl Genome Browser [114], the WashU EpiGenome Browser [115, 116], the NCBI Map Viewer [117] and MEXPRESS [118]. Each browser has a unique interface, set of features and capacity for data integration from external data sources. Here, we cover four browsers tailored to epigenetics data: the UCSC Genome Browser, the WashU EpiGenome Browser, MEXPRESS and cBioPortal.

The UCSC Genome Browser provides access to the genomes of multiple species, including different assemblies. The user can direct the browser to a gene or region, or scroll to browse the genome. The display can be customized to include a wide variety of genome-wide tracks, including genes, structural elements, methylation and acetylation data imported from ENCODE, and many others to enable comparative genomics. It is also possible to include custom tracks either from other public data sets or uploaded by the user [112, 113].

Similar to the UCSC Genome Browser, the WashU EpiGenome Browser includes genome assemblies of several species. The WashU EpiGenome Browser also allows the user to load tracks calling on data from public hubs, as well as custom tracks either uploaded by the user or through a URL link. Publicly available tracks are similar to those offered by other browsers, including genes, structural and genetic variation, repeat masking and others. The WashU EpiGenome Browser also includes a number of epigenomic tracks and chromatin interaction data (e.g. ChIA-PET; HiC). This browser also includes several applications for additional visualization, such as a scatter plot displaying tracks for gene sets provided by the user [115, 116].

MEXPRESS is a Web-based tool that stores all DNA methylation data from TCGA for gene-level analysis. Specifically, this database queries by gene and tumor type and then correlates clinical covariates and expression with measurements of DNA methylation for that gene from TCGA. Analysis is performed across all probes in the array for the query gene to distinguish the impact of epigenetic alterations throughout gene promoters, gene body and CpGIs. cBioPortal also enables analysis of DNA methylation but is limited to analysis of the single probe per gene. In this case, the probe that is most strongly anticorrelated with DNA methylation is selected for analysis. Therefore, cBioPortal does not enable the more complex epigenetic regulation of gene expression facilitated by MEXPRESS [118].

Another widely used genome browser is the Integrative Genomics Viewer (IGV).^1,2^ Similar to other online browsers, IGV can pull, integrate and visualize a wide variety of data from publicly available databases including ENCODE, TCGA, 1000 Genomes and other sources. Users can also provide URLs for data sets or load their own data. IGV can visualize a wide array of data types, and the user can fine-tune and customize the display, and save multiple instances of these settings for future use [119, 120].

## Bioinformatics techniques

For all epigenetic data, be it microarray or next-generation sequencing, the bioinformatics pipeline follows three major steps: (1) quality control, (2) preprocessing and (3) analysis [121]. Typically, each of these steps is performed independently for each study and data modality. The bioinformatics techniques for each of these steps are active areas of research. The maturity of analysis techniques matches the age of each measurement technology. Once each data set is understood independently, epigenetic data can be integrated with other cancer genomics data to determine its functional impact. Such robust, integrated techniques are emerging in bioinformatics. Still, further research in data integration is essential to establish epigenetic drivers of carcinogenesis and therapeutic response for precision medicine.

### DNA methylation normalization and analysis

Preprocessing high-throughput epigenetic data is critical to obtain accurate results. Supplemental File 1 describes techniques for each measurement technology in detail. For microarrrays, preprocessing entails image processing and normalizing probe intensities. Whole-genome bisulfite sequencing (WGBS) techniques require alignment to a reference genome and quantification similar to most second-generation bulk RNA sequencing techniques. As such, most preprocessing pipelines rely on modification of algorithms developed for RNA sequencing. MBD-seq data adapt peak-calling algorithms from ChIP-seq such as MACS [122] to distinguish genomic regions that are methylated. Whereas MBD-seq is nonquantitative, both microarrays and WGBS provide quantitative measurements of the percentage of methylation at each probe or genome coordinate. These estimates can be allele specific for stranded WGBS. Nonetheless, the false-positive rate for MBD-Seq is far lower than arrays or WGBS. As newer technologies, preprocessing techniques for both Nanopore and PacBio methylation analysis are emerging in the literature.

Signal from DNA methylation data can contain technical artifacts independent of the biological conditions, similar to the batch effects established in other high-throughput data sets [123]. These artifacts are most predominant when comparing data across distinct studies but may also be present within large cohort studies such as TCGA. Visualization tools have been developed to assess these technical artifacts from DNA methylation arrays [124, 125] and are an important first step to any analysis of large cohort studies. Many batch correction techniques developed for gene expression microarrays [126, 127] have been applied to DNA methylation arrays [128]. However, care must be taken when applying these algorithms to maintain the distribution of DNA methylation values. Therefore, normalization techniques that account for this distribution [129, 130] and tissue specificity [131] better correct for batch effects in DNA methylation arrays. Similar to gene expression microarrays, batch correction techniques must be selected to preserve signal for the desired analysis [132]. Sequencing-based measurements of DNA methylation are not immune to batch effects. However, these have been less studied than DNA methylation arrays. Gene-level estimates of DNA methylation from bisulfite sequencing could be corrected with standard expression-based techniques, with the same caveats that apply to microarrays. However, these techniques will not extend to locus-specific methylation estimates or DNA methylation from MBD-sequencing. In all cases, obtaining accurate signal requires considering batch effects as part of the experimental design, most especially avoiding perfect confounding between known technical artifacts (e.g. site of tissue source, sampling batch, etc.) and experimental conditions [123].

Many analytic tools for DNA methylation data were developed from well-established procedures for gene expression analysis. Accordingly, techniques for detecting robust differences between two or more conditions are the most ubiquitous and reviewed extensively in [133]. Case-control studies and comparisons of matched tumor and normal tissue from the same individual in cancer epigenetics lend themselves to this type of analysis. Wilcoxon rank‐sum tests and *t-*tests comparing the methylation status of individual genes between two or more groups are the most basic and commonly used analysis. To impact expression, DNA methylation changes must occur over a region of the genome. Therefore, bump-hunting algorithms have also been developed to determine differentially that distinguish sample phenotypes [134]. Variably methylated regions inferred with bump-hunting [134] and outlier-based analysis algorithms [135] are ideally suited to capture inter-tumor heterogeneity in DNA methylation alterations.

Because MBD-seq data obtain calls of methylated regions, alternative statistical methods either comparing the signal relative to input control or comparing binary calls necessary for its analysis. Techniques to compare peaks across conditions are currently emerging in the literature [136, 137]. Peak comparison algorithms are primarily divided into: (1) linear models comparing peaks similar to DMRs, such as DiffBind [138]; (2) hidden Markov models (HMMs), such as ChiPDiff [139] and ChromHMM [140]; or (3) whole-genome correlations, such as StereoGene [141] and GenometriCorr [142]. For all data platforms, more advanced methods using mixture models, Shannon entropy, logistic regression, nonnegative matrix factorization, clustering, feature selection, and correlation are also emerging in the literature.

### Chromatin analysis

ChIP-seq is a pull-down assay similar to MBD-Seq. Therefore, these data use similar preprocessing and differential analysis techniques to those described for MBD-Seq data described above. Robust standards for quality control and preprocessing were adopted by ENCODE as gold standards for all chromatin-based analyses [143]. These preprocessing techniques are also applicable to all other pull-down assays of chromatin structure, including DNaseI-Seq, MNase-Seq, FAIRE-Seq and ATAC-Seq. These preprocessing steps predominately select defined discrete genomic regions that are enriched for the histone mark selected by the antibody used for the ChIP-assay. Determining the chromatin structure specific to cancer cells or cancer subtypes requires differential binding algorithms similar to those described in analysis of MBD-Seq data and reviewed in [136]. In contrast, chromosomal capture methods, i.e. 5C and HiC, produce multiple values representing interaction profiles for each gene. Current bioinformatics tools to preprocess and analyze these data are summarized in [144] and rapidly developing.

All the analysis steps described above define only significant regions in chromatin structure. Further analysis is essential to annotate the function of these genomic regions into various states, including active promoter, weak promoter, poised promoter, strong enhancer, weak/poised enhancers, insulator, transcriptional transition and heterochromatin. ChromHMM is a widely used method to predict chromatin states [140]. This algorithm uses a multivariate HMM to integrate multiple histone modification ChIP-seq data sets. The resulting model can then be used to annotate the chromatin states in a specific cell type. Segway [145] is an unsupervised pattern discovery approach to analyzing chromatin states. The method uses a dynamic Bayesian network model, which is the first genomic segmentation method designed for integration multiple ChIP-seq experiments. Both methods annotate genomes into various states including active promoter, weak promoter, poised promoter, strong enhancer, weak/poised enhancers, insulator, transcriptional transition and heterochromatin. ChromHMM [140] and Segway [145] can both integrate the datasets from histone modification, open chromatin and binding profiles of specific proteins such as CTCF to further establish tissue-specific epigenetic regulation and functional epigenomics relationships.

### Determining functional impacts of epigenetic modifications with data integration

Regardless of the data type, biology defines specific relationships between epigenetic regulation and gene or protein expression. Thus, identifying a functional regulatory role from the resulting epigenetic data requires associating the changes with alterations in gene or protein expression. However, technical heterogeneity and confounding from nonbiological artifacts, such as batch effects [123], library preparation [146] and antibody quality [147], are problematic within a single data type. These technical artifacts can introduce complexities that are prohibitive to integrative analysis in several biological conditions [148].

One complication in integrated analyses is the presence of distinct sources of technical variation in each data modality. To avoid this complexity, many integrated techniques first perform separate analyses data type and then search for associations through the colocalization of significant results at the same genomic location, for example associating hypomethylation of the promoter for a given gene with an increase of gene expression. These methods require matched samples for each set of comparisons a major limitation when dealing with a finite amount of tissue. Additionally, as a given gene in a subtype of cancer is likely to be affected in only a small fraction of individuals, loci-based approaches can be unsuccessful in detecting meaningful biological relationships. Thus, techniques, such as OGSA [149] and RTOPPER [150], seek to increase power and biological inference by integrating these univariate differential results over pathway and genes sets. Algorithms integrating these statistics can be adapted to analyze epigenetic regulation of gene expression from distinct genomics data sets from cohorts with similar study design, and need not necessarily have measurements from the same samples in all data modalities. Outlier-based approaches for this integration, such as OGSA [149], are best suited to capture inter-tumor heterogeneity of epigenetic pathway regulation.

**Figure 4.**
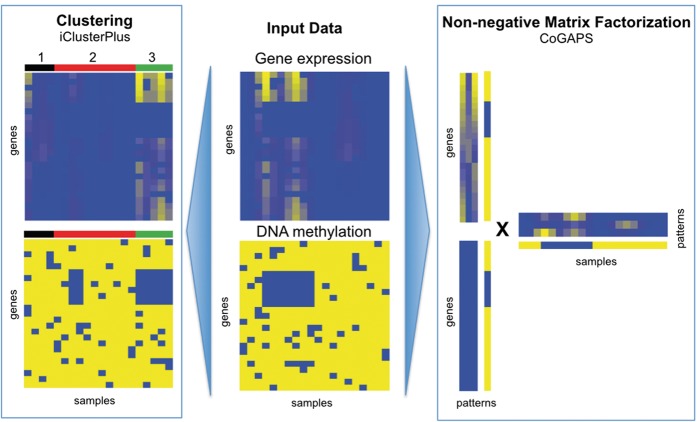
Complete data integration to determine epigenetic regulation of gene expression can be performed for data sets containing both gene expression data (top center) and epigenetic data (bottom center) on the same samples. Clustering-based techniques such as iClusterPlus (left) seek sets of samples that have epigenetic alterations with coordinated gene expression changes. Matrix factorization-based techniques such as CoGAPS (right) infer quantitative relationships between epigenetic alterations and gene expression. These algorithms simultaneously quantify the extent of the coordinated alterations in gene expression and DNA methylation in each sample. Post hoc analyses of the clusters in iClusterPlus or CoGAPS patterns can determine their functional impact in cancer. (A colour version of this figure is available online at: https://academic.oup.com/bfg)

In contrast to gene-based integration analyses, fully integrated analysis has additional power to identify genes or pathways that are often disrupted by multiple mechanisms but at low frequencies by any one mechanism. Unsupervised algorithms, such as iCluster [151], Amaretto [152] and matrix factorization algorithms [153, 154], search for patterns common in these diverse molecular components, regardless of regulatory relationships (Figure 4). The coordinated gene activity in pattern sets (CoGAPS) algorithm finds patterns associated with coordinated DNA methylation and expression changes by encoding a distribution that DNA methylation silences gene expression [154]. Similar models of DNA methylation regulation of gene expression are used to determine genes with a functional impact on cancer subtypes in the MethylMix algorithm [155]. Supervised algorithms overcome this limitation by comparing gene-level associations with phenotype in all measurement platforms [156, 157] or in several measurement platforms for multiple pathway members [149, 150, 158]. However, role of DNA methylation in the gene body is not associated with gene expression silencing, and thus more complex to integrate with these techniques. Therefore, further work is needed to develop robust bioinformatics integration algorithms that encode regulatory relationships between genetic and epigenetic alterations as research refines their interrelationship biologically.

Expanding to the use of biologically driven priors to chromatin data, where different marks or spatial relationships have different regulatory effects, presents additional challenges and may require adapting techniques developed for dealing with multiple targets in microRNA (miRNA) expression to the chromatin landscape [117, 118, 125]. Time course methods such as miRDREM have also been developed to determine the timing of activity of miRNA by integrating their expression with that of mRNA targets [159]. These techniques could readily be adapted to determine the functional impacts of chromatin regulation from time course data of cancer development, metastasis and therapeutic resistance emerging in the literature. Bioinformatics methods are currently being developed that focus on aggregating over epigenetic modifications with similar effects on gene expression, i.e. all repressive or all activating. The ELMER algorithm was developed to incorporate genome-wide maps of enhancers and transcription factors with methylation and expression data to determine epigenetic regulation of transcription factors in cancer [160]. Encoding ways to account to multiple often-conflicting modes of regulation through dysregulation techniques [161] remain a promising avenue for future research.

All the algorithms for integration described above compare gene- or geneset-level summaries of both the epigenetics and genomics data to associate them with phenotypes in cancer. As is the case for CpGIs [24], epigenetic alterations in noncoding regions of the genome have critical functional alterations in cancer. In these cases, associating the genome-wide distribution epigenetic alterations with that of the genome, transcriptome and proteome are essential to determine their functional impact. Both Segway [145] and ChromHMM [140] perform integrated analysis tailored to annotating regions of epigenetic regulation. More general genome-wide correlation techniques can perform additional integration to infer more complex regulatory relationships. GenometriCorr [142], which computes the correlation between sets of genomic intervals, can be used to integrate different types of data—such as the location of gene promoters and transcription factor-binding sites or other annotation. For a pair of interval-represented data sets, GenometriCorr estimates a variety of correlations that are based on interval overlaps, on relative genomic distances and on absolute genomic distances. GenometriCorr is limited to correlation between binary calls along genome tracks and thus requires an interval calling procedure, e.g. MACS [162] to prepare the interval data. In contrast, StereoGene [141] does not require such an identification of intervals. StereoGene uses kernel methods to correlate genome-wide patterns in intensity between two data sets. In this case, epigenetic and genomic profiles may be used as direct inputs for patterns in StereoGene to correlate levels of epigenetic regulation. Both algorithms compute pairwise comparisons between genomic profiles or linear combination of profiles. Therefore, the integration of numerous data types from many samples cannot be computed directly. Instead, these pairwise correlations (or distances) could be inputs to other supervised or unsupervised analysis techniques to infer common epigenetic regulation across cancer samples. Future research into methods that perform genome-wide coordinate-based integration techniques across multiple samples is essential to determine the full impact of epigenetic alterations on functional genomic alterations in cancer.

## Discussion

Elucidating the relationships between different epigenetic mechanisms and their regulation of gene expression is essential to finding hidden sources of variation in cancer and therapeutic selection. New high-throughput measurement technologies enable unprecedented, quantitative measurements of the epigenetic state in cancers. For DNA methylation, these techniques can be applied readily to both primary tumors and model organisms. Therefore, the functional impact of methylation alterations can be assessed bioinformatically in targeted experiments on model organisms and across sample population. On the other hand, chromatin assays require higher-quality and quantity samples that are typical not feasible for preserved tumor samples or biopsies. As a result, chromatin measurements are typically limited to model organisms. In the case of DNA methylation, the epigenetic landscape of cell line models has been shown to vary significantly from that of primary tumors relative to PDXs [163]. We anticipate similar discrepancies between model organisms and primary tumors in the chromatin landscape. Thus, advances that adapt chromatin measurement techniques to primary tumor samples are essential to cancer epigenetics.

Databases with epigenetic and genomic data, such as TCGA, ENCODE and FANTOM, are an important step toward achieving this goal. Individually, these large public domain data sets have fueled algorithm development and understanding of epigenetic and tumor-based gene expression changes, respectively. While TCGA contains DNA methylation, gene expression and proteomic data in thousands of primary tumors across cancer types, it lacks chromatin data. Chromatin and transcriptional data are available for numerous cancer cell lines in ENCODE and FANTOM, but these databases lack DNA methylation data and data from primary tumors. However, interactions between DNA methylation and chromatin structure are essential in functional epigenetic regulation. Therefore, it is essential to develop a comprehensive database of matched epigenetic, genetic and phenotypic data.

A comprehensive catalog is especially important for primary tissue samples, where intra-tumor heterogeneity compounds the effect of inter-individual heterogeneity further obscuring the underlying drivers of disease. Many resistance mechanisms to therapeutics are associated with tumor heterogeneity [164, 165]. Advances to single-cell genomic and epigenomic techniques in recent years enable quantification of the heterogeneity of epigenetic changes in cancer progression and therapeutic response. These single-cell techniques are also able to measure epigenetics in samples with low cell count analysis, facilitating analysis of tumor and biopsy samples. However, similar to chromatin, they are limited to fresh tissues. New methods for single-cell Hi-C [166], scATAC-Seq [89], scChIP-Seq [167] and scBS-Seq [168] are quickly being refined and have opened the door for querying the epigenome at all levels. Furthermore, the inherent disadvantages from their original approaches are not eliminated and can even be amplified with additional complications related to adequate single-cell isolation and poor sequencing resolution [169, 170]. Advancements in fluorescence-activated cell sorting (FACS), microchip arrays and microfluidics have made high-throughput cell isolation more tenable, but significant price limitations and bias toward certain cell sizes (microfluidics), cell markers (FACS) or rare cell types (microchip) remain [170, 171].

Numerous bioinformatics techniques have been developed to preprocess and analyze single-platform data for DNA methylation and chromatin structure. However, establishing a functional link in cancer requires further identification of epigenetic alterations that are associated with gene expression, protein and phenotypic changes. Integrating data across measurement platforms is essential to establish these functional relationships. To date, most of these techniques are limited to correlations between genes or common clusters shared across data sets. New integrated bioinformatics techniques are essential to model and distinguish different forms of epigenetic regulation in driving tumor heterogeneity and ultimately cancer. While integrated analyses are emerging, few tools are designed to encode and test these regulatory relationships directly. Determining the true epigenetic regulatory mechanisms and drivers of cancer pathology will be essential for precision medicine with emerging epigenetic therapies.

Key Points

- Epigenetic alterations compliment genomic alterations during cancer progression and therapeutic response.
- High-throughput measurement technologies can characterize the epigenetic landscape of tumors and model organisms.
- Epigenetic data in large panels of human tumors and cell lines are available from large research consortium.
- Bioinformatics algorithms that integrate epigenetic data with genomics data are essential to determine the function of epigenetic alterations in cancer.

## Supplementary data

Supplementary data are available online at https://academic.oup.com/bfg.

## Funding

This work was supported by National Institutes of Health (NIH) Grants R01CA177669, R21DE025398, P30 CA006973, and Specialized Programs of Research Excellence in Human Cancers (SPORE) P50DE019032.

## Supplementary Material

Supplementary Data

## Notes

**Luciane T. Kagohara** is a Postdoctoral Research Fellow in the Department of Oncology Johns Hopkins University SKCCC. She studies the role of aberrant epigenetic and gene expression markers in cancer.

## Notes

**Genevieve Stein-O'Brien** is a PhD student at the McKusick-Nathans Institute of Genetic Medicine, Johns Hopkins Medical School. Her research focuses on human development and construction of analytical tools for genetic and epigenetic analysis.

## Notes

**Dylan Kelley** is a Researcher in the Department of Otolaryngology, Johns Hopkins University. He is investigating chromatin modification and alternative splicing in transcriptional regulation of head and neck cancer.

## Notes

**Emily Flam** is a Researcher in the Department of Otolaryngology, Johns Hopkins University. She is studying the role of enhancers and methylation in transcriptional regulation of head and neck cancer.

## Notes

**Heather C. Wick** is a PhD Candidate at the Institute of Genetic Medicine at Johns Hopkins University School of Medicine. She is studying transcriptional programs and chromosomal remodeling in prostate cancer.

## Notes

**Ludmila V. Danilova** is a Research Associate in the Department of Oncology Johns Hopkins University SKCCC. Her primary interest is in integrative analysis of different types of cancer genomics data.

## Notes

**Hariharan Easwaran** is an Assistant Professor in the Department of Oncology Johns Hopkins University SKCCC. He investigates the mechanisms and roles of epigenetic alterations in cancer etiology.

## Notes

**Alexander V. Favorov** is a Research Associate in Johns Hopkins University SKCCC and a Senior Researcher at VIGG RAS and GosNIIGenetika. He develops statistical approaches to decipher genome-scale data.

## Notes

**Jiang Qian** is a Professor in the Wilmer Eye Institute, Johns Hopkins University. He works on computational analysis of genetic and epigenetic regulatory networks in various systems.

## Notes

**Daria A. Gaykalova** is an Assistant Professor in the Department of Otolaryngology, Johns Hopkins University. She investigates the role epigenetics in transcriptional regulation of head and neck squamous cell carcinoma progression.

## Notes

**Elana J. Fertig** is an Assistant Professor in the Department of Oncology Johns Hopkins University SKCCC. She develops bioinformatics pattern detection algorithms for epigenetic and genomic data integration in cancer.
