# Supplementary material for: Epigenetic regulation of gene expression in cancer: techniques, resources and analysis
Source: Brief Funct Genomics. 2017 Aug 11;17(1):49–63. doi: 10.1093/bfgp/elx018 (PMC5860551; doi:10.1093/bfgp/elx018)
Supplement: Supplementary Data [file elx018_supplementalmaterials_ltk_ejf.docx]

**DNA Methylation**

Array hybridization

Genome wide measurements of DNA methylation were first enabled through microarrays [1], with platforms such as the Illumina GoldenGate BeadArray that measure specific CpG sites in genes with established functions in cancer. The DNA is treated with sodium bisulfite, which converts unmethylated cytosines to uracils and leaves methylated cytosines unchanged. After bisulfite conversion and DNA amplification, the converted uracils are detected as thymine and the methylated sites as unchanged cytosines [2,3]. The DNA microarrays using primers for the query bisulfite treated DNA and the input native DNA. The two probes (methylated and unmethylated) are labeled with different dyes and the products are hybridized to the beads representing CpG sites and the signal is measured.

In DNA methylation arrays, the methylation level of a specific CpG (β) can be quantified from the ratio of intensity in the methylated (M) and unmethylated (U) dyes as . As with all arrays, DNA methylation arrays require normalization. First image processing is used to compute probe intensities using vendor-provided software with the most common being Illumina BeadScan software for the widely used Infinnium 450K Arrays. The processed probe intensities are then normalized to minimize technical bias when inferring absolute DNA methylation levels. Normalization pipelines are incorporated in the commercially available software from the array companies. A number of algorithms accounting for the distribution of DNA methylation values and probe chemistry have also been developed [4–9]. Many of these algorithms are included in the R/Bioconductor packages for Illumina arrays such as lumi [10], minfi [11], and Methylumi.

DNA methylation analysis using bisulfite-converted DNA requires special characteristics such as probes based on the converted sequence or arrays that are more flexible to mismatches during hybridization [12,13]. The mismatches were a major problem for the first DNA methylation arrays [14,15], since poorly methylated regions generated low hybridization signals due to C->U conversion if compared to densely methylated portions that were mainly intact and less affected by mismatch due to the mCpG presence. Microarray platforms have been advancing the technology of probes to address cross binding and increase the density of measurements of DNA methylation on the genome beyond CpG island promoter regions. Today, platforms such as the Infinium HumanMethylation850 Bead Chip Array from Illumina (Illumina 850K), Human CpG Island Microarray Kit (Agilent) and GeneChip Human Promoter 1.0R Arrays (Affymetrix), have developed to overcome hybridization issues and represent good alternatives to study the CpG sites throughout the genome with high resolution. Arrays are a sensitive and well-established method for methylation profile analysis in human cancer and other diseases [12,13]. Although thousands of genes can be analyzed, the CpG sites are limited to the ones present in the array chip design.

Whole Genome Bisulfite Sequencing (WGBS)

WGBS allows the interrogation of methylation status of individual CpGs but in a genome-wide scale. Similar to microarrays the target DNA is bisulfite converted. Both bisulfite converted DNA and untreated input controls are sequenced with high throughput next generation sequencing technologies. In contrast to arrays, this technique enables whole genome interrogation of DNA methylation and strand-specific assessment of methylation status. However, alignment of the bisulfite treated DNA to the genome is critical to the accuracy of this method. Bisulfite conversion reduces genome complexity from four nucleotide types to three, except for the few methylated CpG sites, in many cases making alignment to the reference genome non-unique and introducing errors into the quantification [12,13]. Therefore, new bioinformatics techniques for preprocessing WGBS remain a critical challenge preceding the analysis of WGBS data. Short read aligners such as Bowtie [16], BWA [17], and STAR [18] can be modified to account for the selective depletion of unmethylated cytosines (Cs) for bisulfite sequencing. Additionally, two alternative approach for bisulfite sequencing have been developed to take advantage of the enrichment of DNA methylation at CpGs in the human genome. Wild‐card aligners modify the alignment scoring matrix to prevent mismatch penalties between Cs and Ts resulting from the bisulfite‐induced conversion [19–28]. Three‐letter aligners reduce this process further by simply replacing all Cs with Ts and carrying out standard alignment on a three (vs four) base genome [29–32]. Despite differences in speed and accuracy, aligner choice has been shown to have a little impact on the platform comparisons [33]. Thus, primary considerations for potential bias and resolution in epigenetic data should be addressed at the platform level prior to collection.

Methyl-CpG binding domain protein-enriched genome sequencing (MBD-seq)

Just like WGBS recently developed MBD-Seq provides a probe-independent genome-wide evaluation of the methylome landscape. While resolution of the MBD-Seq is lower than in WGBS, MBD-Seq has several advantages that result in greater accuracy of whole-genome DNA methylation detection. MDB-Seq utilizes unconverted DNA, and therefore it does not depend on the efficiency of the intermediate steps, such as bisulfite conversion. Methyl-binding domain of MBD2 (MBD2-MBD) protein has nanomolar affinity for a single symmetrically methylated CpG dinucleotide. Conversely, the MBD2-MBD does not bind unmethylated DNA oligonucleotides to any appreciable extent. The massively parallel next generation sequencing of MBD-captured DNA fragments provides quantitative whole-genome evaluation of DNA methylation and high resolution allows estimating a single mCpG site [34,35].

MBD-seq data provides two sets of reads, (1) methylated regions determined from pull-down and (2) input control. The number of reads corresponding to any given genomic DNA segment detected by MBD-Seq relative to the number of reads for an input control sample is proportional to the number of methylated CpG dinucleotides across all DNA fragments from that region. Therefore, this data is similar to ChIP-seq data. As with bisulfite sequencing, alignment of both sets of reads is a critical first step to analysis. Once aligned, regions of the genome that are methylated are determined from regions with large read counts in the methylated signal relative to input control. Peak calling algorithms such as MACS [36] are a particularly popular set of enrichment-based methods for this problem, developed first for ChIP-seq data. Drawing from the well-established statistical approaches for differential analysis, many peak calling algorithms rely on models derived from negative controls to call enrichment.

# **Chromatin Structure**

ChIP-Seq

DNA accessibility assays lack the information about the nature of DNA binding proteins [37], which can be evaluated by Chromatin immunoprecipitation (ChIP)-based methodology. ChIP has been used to study association of any proteins including TFs, enzymes, or histones and their modified isoforms with genomic DNA [38,39]. Briefly, ChIP isolates regions of DNA bound for the protein of interest, recognized by specific antibodies. Arrays or sequencing are used to determine the sequence of these DNA fragments bound by the study histones, transcription factors or enzymes. Peak callers such as MACS [36] are used to determine DNA regions with significant protein enrichment relative to input control. Robustness of these peak calls can be assessed through replicates, using protocols established for the ENCODE consortium[40]. Similar bioinformatics techniques are employed to process chromatin structure for all the techniques described in the remainder of this section.

ChIP-seq uses antibodies to that bind to the specific protein or its modification of interest for selection and DNA enrichment [41,42]. Therefore, this methodology highly depends on the quality and specificity of the utilized antibodies. ChIP-seq can also be performed for both activating and repressive histone marks. Even upon proper chromatin digestion condensed chromatin with repressive marks is under-digested, resulting in poor amplification of longer DNA segments during library preps for the sequencing, and poor resolution of repressive histone mark mapping upon genome alignment of these long fragments of DNA associated with such heterochromatin. While ChIP-Seq analysis provides information about whole-genome distribution of individual proteins or their modifications – individual samples should be prepared for each study proteins, and therefore analysis of several proteins can be costly and require high material input.

DNaseI-Seq

As mentioned earlier, chromatin is the DNA-protein complex that compacts and protects the genomic DNA within the cellular nucleus and the carrier of epigenetic information. Therefore, the structure of chromatin can be evaluated by DNA accessibility to the restriction reagents, such as DNaseI, which recognizes DNase hypersensitivity sites (DHSs). The chromatin structure decreases the accessibility of such regions to DNaseI digestion. High-throughput sequencing, or chip-annealing, of DNaseI pre-treated DNA reveals whole-genome structure of the chromatin in individual samples and define regions with open chromatin in functionally active regulatory elements such as promoters, silencers, enhancers, as well as intergenic regions [43–45]. Due to the sequence-dependence – DNase-Seq has moderate resolution. One limitation of DNase-Seq analysis shared by all high-throughput chromatin techniques includes under-digested chromatin (DNA fragments > 900bp) that is poorly amplified and underrepresented during sequences or probe annealing. Moreover, individual samples have different cellular properties, and the degree of chromatin digestion must be empirically determined for individual samples. The chromatin integrity and DNA-protein binding strength highly depend on sample preservation and affects the rate of digestion.

MNase-Seq

MNase-Seq follows the same procedure as DNase-Seq, replacing the restriction reagent with MNase. The main advantage of MNase is that it does not depend on DNA sequence or digest all of accessible nucleosome-free DNA that helps to define the high-resolution nucleosome positioning, and therefore the chromatin structure genome-wide. However, MNase has higher preference for A/T-reached DNA and can over-digest even nucleosomal DNA at longer incubation Over-digestion of the chromatin by MNase results in nucleosome disruption and DNA loss during the library enrichment step. Nonetheless, MNase-Seq has high resolution for high sample input [46].

## FAIRE-Seq

Formaldehyde-Assisted Isolation of Regulatory Elements Sequencing (FAIRE-Seq) is a method for the detection of nucleosome-free DNA similar to DNase- or MNase-Seq. In FAIRE-Seq, the chromatin is broken by sonication, which introduce the random double-strand breaks throughout the genome. Phenol extraction allows protein-bound DNA, such as nucleosomes, to remain in the organic phase, while protein-free DNA fragments are extracted to the aquatic phase. The advantage of this technique is independence of chromatin digestion from restriction reagents as well as buffer composition [47]. Nonetheless, it has low resolution similar to DNase-Seq.

ATAC-Seq

ATAC-Seq is a relatively new approach that uses the ability of Tn5 transposase to cut and ligate adapters to the genomic DNA. Tn5 can access and integrate adaptors in regions of open chromatin, while transposition in non-accessible sites is unlikely to occur. Therefore, ATAC-seq can be used to characterize nucleosome positioning by size and periodicity of Tn5 adaptors inserted [37]. Another advantage of this genome-wide approach is the small amount of input material, on the order of a few thousand cells, 10,000-fold less than DNase-Seq, MNase-Seq, or ChIP-Seq. Often, these high demands for sample input are unachievable for tumor samples due to chromatin structure disruption throughout the procedure, material loss over the enrichment/purification steps, and overall low DNA outcome/output [48,49]. Therefore, ATAC-seq is a particularly promising technique on which to profile the chromatin structure on samples inaccessible to other chromatin-based assays.

# **Interaction of chromatin domains**

## Chromosome conformation capture (Hi-C)

Chromosome conformation capture can be used to infer the three-dimensional structure of the genome. The process generates fragments of cross-linked DNA by using formaldehyde followed by chromatin fragmentation by restriction enzymes. Cross-linked DNA fragments are linked together by ligation through the sticky ends and amplified after cross-link reversion. Early chromosome conformation capture were performed in low throughput: 3C and 4C approaches designed to query specified DNA-cross links [50–52]**.** The chromosome conformation capture carbon copy (5C) approach enables high throughput interrogation with arrays, but is not a genome-wide approach [51,53]. Hi-C is the newest of the 3C techniques. During the 3C library preparation, the interaction fragments are conjugated with biotin before the ligation step. After cross-link reversion, the biotinylated fragments are captured by streptavidin in an unbiased way. The target fragments are then identified by paired end sequencing [54].

Preprocessing techniques for Hi-C data are reviewed extensively in [55]. Briefly, reads for a pair of interacting genomic regions are aligned to the genome and quantified. Instead of providing a single locus per sample as in other techniques, this preprocessing provides a quantitative profile for interactions between genomics coordinates in a single sample for bioinformatics analysis. Because these data represent the sum of interactions across a large number of cells, the majority of interactions are assumed to be nonspecific, leading to a model of baseline interaction frequency as a function of genomic distance [56]. Analytical approaches can be divided by the method used to power detection above this baseline. Looping interactions identify pairs of loci that interact more frequently than expected while topologically associating domains (TADs) rely on increased interactions across multiple loci within a specific region. Alignment and analysis techniques for Hi-C data are currently an active area of research in genomics.

## Chromatin Interaction Analysis by Paired-End Tag (ChIA-PET)

ChIA-PET Sequencing is a combined technique that makes use of both 3C and ChIP-Seq techniques. DNA is cross-linked and immunoprecipitated against the protein of interest and the 3C protocol is performed, followed by NGS to identify the fragments from the immunoprecipitation [57]. As a result, this data measures DNA interaction domain with specific protein binding.

**References**

1. Yan PS, Chen CM, Shi H, et al. Dissecting complex epigenetic alterations in breast cancer using CpG island microarrays. Cancer Res. 2001; 61:8375–8380

2. Frommer M, McDonald LE, Millar DS, et al. A genomic sequencing protocol that yields a positive display of 5-methylcytosine residues in individual DNA strands. Proc. Natl. Acad. Sci. U. S. A. 1992; 89:1827–1831

3. Mill J, Petronis A. Profiling DNA Methylation from Small Amounts of Genomic DNA Starting Material: Efficient Sodium Bisulfite Conversion and Subsequent Whole-Genome Amplification. DNA Methylation 2009; 507:371–391

4. Teschendorff AE, Marabita F, Lechner M, et al. A beta-mixture quantile normalization method for correcting probe design bias in Illumina Infinium 450 k DNA methylation data. Bioinformatics 2013; 29:189–196

5. Maksimovic J, Gordon L, Oshlack A. SWAN: Subset-quantile Within Array Normalization for Illumina Infinium HumanMethylation450 BeadChips. Genome Biol. 2012; 13:R44

6. Assenov Y, Müller F, Lutsik P, et al. Comprehensive analysis of DNA methylation data with RnBeads. Nat. Methods 2014; 11:1138–1140

7. Fortin J-P, Labbe A, Lemire M, et al. Functional normalization of 450k methylation array data improves replication in large cancer studies. Genome Biol. 2014; 15:

8. Oros Klein K, Grinek S, Bernatsky S, et al. funtooNorm: an R package for normalization of DNA methylation data when there are multiple cell or tissue types. Bioinformatics 2016; 32:593–595

9. Fortin J-P, Triche TJ, Hansen KD. Preprocessing, normalization and integration of the Illumina HumanMethylationEPIC array with minfi. Bioinformatics 2016; btw691

10. Du P, Kibbe WA, Lin SM. lumi: a pipeline for processing Illumina microarray. Bioinformatics 2008; 24:1547–1548

11. Aryee MJ, Jaffe AE, Corrada-Bravo H, et al. Minfi: a flexible and comprehensive Bioconductor package for the analysis of Infinium DNA methylation microarrays. Bioinformatics 2014; 30:1363–1369

12. Laird PW. Principles and challenges of genomewide DNA methylation analysis. Nat. Rev. Genet. 2010; 11:191–203

13. Kurdyukov S, Bullock M. DNA Methylation Analysis: Choosing the Right Method. Biology 2016; 5:3

14. Adorján P, Distler J, Lipscher E, et al. Tumour class prediction and discovery by microarray-based DNA methylation analysis. Nucleic Acids Res. 2002; 30:e21

15. Gitan RS, Shi H, Chen C-M, et al. Methylation-specific oligonucleotide microarray: a new potential for high-throughput methylation analysis. Genome Res. 2002; 12:158–164

16. Langmead B, Trapnell C, Pop M, et al. Ultrafast and memory-efficient alignment of short DNA sequences to the human genome. Genome Biol. 2009; 10:R25

17. Li H, Durbin R. Fast and accurate short read alignment with Burrows-Wheeler transform. Bioinforma. Oxf. Engl. 2009; 25:1754–1760

18. Dobin A, Davis CA, Schlesinger F, et al. STAR: ultrafast universal RNA-seq aligner. Bioinforma. Oxf. Engl. 2013; 29:15–21

19. Wu TD, Watanabe CK. GMAP: a genomic mapping and alignment program for mRNA and EST sequences. Bioinformatics 2005; 21:1859–1875

20. Wu TD, Nacu S. Fast and SNP-tolerant detection of complex variants and splicing in short reads. Bioinformatics 2010; 26:873–881

21. Xi Y, Li W. BSMAP: whole genome bisulfite sequence MAPping program. BMC Bioinformatics 2009; 10:232

22. Frith MC, Mori R, Asai K. A mostly traditional approach improves alignment of bisulfite-converted DNA. Nucleic Acids Res. 2012; 40:e100

23. Coarfa C, Milosavljevic A. Pash 2.0: scaleable sequence anchoring for next-generation sequencing technologies. Pac. Symp. Biocomput. Pac. Symp. Biocomput. 2008; 102–113

24. Coarfa C, Yu F, Miller CA, et al. Pash 3.0: A versatile software package for read mapping and integrative analysis of genomic and epigenomic variation using massively parallel DNA sequencing. BMC Bioinformatics 2010; 11:572

25. Smith AD, Chung W-Y, Hodges E, et al. Updates to the RMAP short-read mapping software. Bioinforma. Oxf. Engl. 2009; 25:2841–2842

26. Smith AD, Xuan Z, Zhang MQ. Using quality scores and longer reads improves accuracy of Solexa read mapping. BMC Bioinformatics 2008; 9:128

27. Xi Y, Bock C, Muller F, et al. RRBSMAP: a fast, accurate and user-friendly alignment tool for reduced representation bisulfite sequencing. Bioinformatics 2012; 28:430–432

28. Otto C, Stadler PF, Hoffmann S. Fast and sensitive mapping of bisulfite-treated sequencing data. Bioinformatics 2012; 28:1698–1704

29. Krueger F, Andrews SR. Bismark: a flexible aligner and methylation caller for Bisulfite-Seq applications. Bioinformatics 2011; 27:1571–1572

30. Harris EY, Ponts N, Levchuk A, et al. BRAT: bisulfite-treated reads analysis tool. Bioinformatics 2010; 26:572–573

31. Chen P-Y, Cokus SJ, Pellegrini M. BS Seeker: precise mapping for bisulfite sequencing. BMC Bioinformatics 2010; 11:203

32. Pedersen B, Hsieh T-F, Ibarra C, et al. MethylCoder: software pipeline for bisulfite-treated sequences. Bioinformatics 2011; 27:2435–2436

33. Harris RA, Wang T, Coarfa C, et al. Comparison of sequencing-based methods to profile DNA methylation and identification of monoallelic epigenetic modifications. Nat. Biotechnol. 2010; 28:1097–1105

34. Serre D, Lee BH, Ting AH. MBD-isolated Genome Sequencing provides a high-throughput and comprehensive survey of DNA methylation in the human genome. Nucleic Acids Res. 2010; 38:391–399

35. Yegnasubramanian S, Wu Z, Haffner MC, et al. Chromosome-wide mapping of DNA methylation patterns in normal and malignant prostate cells reveals pervasive methylation of gene-associated and conserved intergenic sequences. BMC Genomics 2011; 12:313

36. Feng J, Liu T, Qin B, et al. Identifying ChIP-seq enrichment using MACS. Nat. Protoc. 2012; 7:1728–1740

37. Buenrostro JD, Giresi PG, Zaba LC, et al. Transposition of native chromatin for fast and sensitive epigenomic profiling of open chromatin, DNA-binding proteins and nucleosome position. Nat. Methods 2013; 10:1213–1218

38. Ho JWK, Bishop E, Karchenko PV, et al. ChIP-chip versus ChIP-seq: lessons for experimental design and data analysis. BMC Genomics 2011; 12:134

39. O’Geen H, Echipare L, Farnham PJ. Using ChIP-seq technology to generate high-resolution profiles of histone modifications. Methods Mol. Biol. Clifton NJ 2011; 791:265–286

40. Landt SG, Marinov GK, Kundaje A, et al. ChIP-seq guidelines and practices of the ENCODE and modENCODE consortia. Genome Res. 2012; 22:1813–1831

41. Schmidt D, Wilson MD, Spyrou C, et al. ChIP-seq: using high-throughput sequencing to discover protein-DNA interactions. Methods San Diego Calif 2009; 48:240–248

42. Bentley DR, Balasubramanian S, Swerdlow HP, et al. Accurate whole human genome sequencing using reversible terminator chemistry. Nature 2008; 456:53–59

43. Heintzman ND, Hon GC, Hawkins RD, et al. Histone modifications at human enhancers reflect global cell-type-specific gene expression. Nature 2009; 459:108–112

44. Dixon JR, Selvaraj S, Yue F, et al. Topological domains in mammalian genomes identified by analysis of chromatin interactions. Nature 2012; 485:376–380

45. Hon GC, Hawkins RD, Caballero OL, et al. Global DNA hypomethylation coupled to repressive chromatin domain formation and gene silencing in breast cancer. Genome Res. 2012; 22:246–258

46. Meyer CA, Liu XS. Identifying and mitigating bias in next-generation sequencing methods for chromatin biology. Nat. Rev. Genet. 2014; 15:709–721

47. Giresi PG, Kim J, McDaniell RM, et al. FAIRE (Formaldehyde-Assisted Isolation of Regulatory Elements) isolates active regulatory elements from human chromatin. Genome Res. 2007; 17:877–885

48. Pott S, Lieb JD. Single-cell ATAC-seq: strength in numbers. Genome Biol. 2015; 16:172

49. Buenrostro JD, Wu B, Litzenburger UM, et al. Single-cell chromatin accessibility reveals principles of regulatory variation. Nature 2015; 523:486–490

50. Dekker J, Rippe K, Dekker M, et al. Capturing chromosome conformation. Science 2002; 295:1306–1311

51. Barutcu AR, Fritz AJ, Zaidi SK, et al. C-ing the Genome: A Compendium of Chromosome Conformation Capture Methods to Study Higher-Order Chromatin Organization: CHROMOSOME CONFORMATION CAPTURE METHODS. J. Cell. Physiol. 2016; 231:31–35

52. Simonis M, Klous P, Splinter E, et al. Nuclear organization of active and inactive chromatin domains uncovered by chromosome conformation capture-on-chip (4C). Nat. Genet. 2006; 38:1348–1354

53. Dostie J, Richmond TA, Arnaout RA, et al. Chromosome Conformation Capture Carbon Copy (5C): a massively parallel solution for mapping interactions between genomic elements. Genome Res. 2006; 16:1299–1309

54. Lieberman-Aiden E, van Berkum NL, Williams L, et al. Comprehensive mapping of long range interactions reveals folding principles of the human genome. Science 2009; 326:289–293

55. Schmitt AD, Hu M, Ren B. Genome-wide mapping and analysis of chromosome architecture. Nat. Rev. Mol. Cell Biol. 2016; 17:743–755

56. Dekker J, Marti-Renom MA, Mirny LA. Exploring the three-dimensional organization of genomes: interpreting chromatin interaction data. Nat. Rev. Genet. 2013; 14:390–403

57. Fullwood MJ, Ruan Y. ChIP-based methods for the identification of long-range chromatin interactions. J. Cell. Biochem. 2009; 107:30–39
